# New Insights into the Role of Histone Changes in Aging

**DOI:** 10.3390/ijms21218241

**Published:** 2020-11-03

**Authors:** Sun-Ju Yi, Kyunghwan Kim

**Affiliations:** Department of Biology, School of Biological Sciences, College of Natural Sciences, Chungbuk National University, Cheongju 28644, Chungbuk, Korea; sjyi@chungbuk.ac.kr

**Keywords:** aging, histone level, histone modification, histone variant, chromatin remodeling, epigenetics

## Abstract

Aging is the progressive decline or loss of function at the cellular, tissue, and organismal levels that ultimately leads to death. A number of external and internal factors, including diet, exercise, metabolic dysfunction, genome instability, and epigenetic imbalance, affect the lifespan of an organism. These aging factors regulate transcriptome changes related to the aging process through chromatin remodeling. Many epigenetic regulators, such as histone modification, histone variants, and ATP-dependent chromatin remodeling factors, play roles in chromatin reorganization. The key to understanding the role of gene regulatory networks in aging lies in characterizing the epigenetic regulators responsible for reorganizing and potentiating particular chromatin structures. This review covers epigenetic studies on aging, discusses the impact of epigenetic modifications on gene expression, and provides future directions in this area.

## 1. Introduction

An individual organism undergoes a series of developmental stages, including birth, growth, maturity, aging, and death. Aging is the gradual and continuous decline or loss of function at the cellular, tissue, and organismal levels with the passage of time. Aging is considered to begin in early adulthood, but is regarded as an integral part of life since other stages also affect the aging process [1]. Aging itself is regarded as a risk factor for numerous chronic diseases, such as neurodegenerative diseases, cardiovascular diseases, metabolic diseases, and osteoporosis. Due to an increase in average life expectancy and a decline in fertility, the global population is currently aging rapidly. Understanding the mechanisms of aging, longevity, and susceptibility to age-related disease is necessary to slow down the rate of aging and increase healthy lifespan [2]. Large numbers of studies have been performed to unravel why and how aging occurs; these studies have suggested that aging is regulated by complex cellular and molecular mechanisms at different developmental stages [1,3]. Numerous factors affecting the aging process and longevity have been reported. Genetic differences and genetic damage are widely known to play an important role in aging, but non-genetic factors such as stress, dietary manipulation, sexual stimuli, circadian rhythms, and exercise have also been implicated [4,5,6,7,8,9]. In particular, social insects such as ants and honey bees have emerged as models to show the influence of environmental stimuli on lifespan. Individuals with a common genome present caste-specific differences in morphology, behavior, and longevity that are determined by environmental inputs early in the developmental process. Recently, environmental cues have been shown to stimulate epigenetic changes, such as DNA methylation and histone modifications, which play critical roles in regulating caste-based differences in longevity and behavior [10,11].

Chromatin is a nucleosome polymer composed of DNA and histone protein. It is a flexible and dynamic structure that exists either as heterochromatin or euchromatin. The former is compact and transcriptionally inactive, while the latter is a decondensed type of chromatin and is transcriptionally active [12,13]. Since the accessibility of transcription regulatory machinery to chromatin is affected by its state, chromatin remodeling is required to expose or hide regions of DNA for transcription [14]. The chromatin structure is remodeled via several mechanisms, including histone modification, histone tail cleavage, exchange of histone variants, and ATP-dependent chromatin remodeling [15,16]. The state of chromatin can be altered by environmental stimuli, which subsequently affects the expression of genes associated with aging and longevity [4,8]. In this review, we discuss recent research focusing on the roles of histone protein levels, the exchange of histone variants, histone modifications, and chromatin remodeling in aging. We also review recent genome-wide profiling of chromatin accessibility patterns and histone modification in young and aged models. Links between chromatin architecture and aging may provide strategies to counter or reverse the aging process or age-related diseases.

## 2. Histone Changes

### 2.1. Histone Levels and Nucleosome Occupancy

Global loss of canonical histones is regarded as a common feature of aging in a range of organisms from yeast to humans (Table 1). In yeast, a decline in canonical histone occurs during replicative aging as a consequence of reduced histone protein synthesis, and ectopic expression of histones H3 and H4 strongly promotes replicative lifespan extension [17]. Micrococcal nuclease digestion followed by sequencing revealed ≈50% nucleosome loss across the whole genome and a fuzzy redistribution of the remaining nucleosomes during replicative aging, increasing the transcription of many genes and resulting in genomic instability [18]. In senescent human fibroblasts, a reduction in histone biosynthesis or lysosomal-mediated processing caused the depletion of histones [19,20]. One report notes that the transcription of histones decreases in quiescent muscle stem cells during chronological aging [21]. Interestingly, Yu et al. demonstrated that reducing the dosage to 85% of wild-type H3 and H4 by deleting the minor H3-H4-coding gene pair *HHT1-HHF1* profoundly extends replicative lifespan, whereas reducing the dosage to 15% by deleting *HHT2-HHF2* shortens the lifespan of yeast. The authors also showed that moderate reductions in H3-H4 activate a distinct stress response and block TOR signaling, thus promoting longevity [22]. However, it remains unclear whether moderate chromatin architectural defects promote longevity in other organisms. Recently, Chen et al. reanalyzed data on H3 chromatin immunoprecipitation and high-throughput sequencing (ChIP-seq) datasets derived from mouse tissues at three different stages of the mouse lifespan. They found a number of age-related changes in H3 occupancy, including increased and decreased H3 occupancy, but no dramatic changes in H3 expression levels [23]. However, the replacement of canonical histone H3 with histone H3 variants, such as H3.3, may occur during aging in mice since the H3 antibody used in these studies recognizes both canonical and variant H3 proteins (see the section of histone variants). Taken together, the results of these studies show that it is likely that histone loss or nucleosome loss occurs in a cell type-specific or context-specific manner during aging.

### 2.2. Histone Variants

Histone variants are isoforms of histones, which have different primary sequences and specialized functional properties compared to canonical histones. Histone variants are expressed throughout the cell cycle and incorporated into chromatin in a replication-independent manner, although canonical histones are synthesized and preferentially incorporated into nucleosomes in a replication-dependent manner. The exchange of canonical histones with histone variants modulates the properties of the nucleosome, thereby affecting DNA replication and transcription [33]. A series of studies implicates histone variants in aging (Table 1). It was found that the level of macro H2A (mH2A) increased in human fibroblasts during replicative senescence. An upregulated level of mH2A was also observed in the liver and lung tissue of aged mice and in the muscle tissue of aged baboons [25]. In human fibroblasts, upregulation of H2A.Z and H3.3 as well as downregulation of H2A.1 and H3.1 was found during aging [24].

Using mass spectrometry, age-related H3.3 accumulation was observed in mouse neurons and postmortem human brains [26]. H3.3 accounts for only a small portion of the total H3 pool in embryonic neuronal chromatin, but in aged mice, H3.3 becomes dominant in the total H3 pool of neuronal chromatin, which in turn regulates cell type-specific gene expression. In mouse somatic tissues such as the liver, kidney, brain, and heart, histone variant H3.3 replaces canonical H3.1 and H3.2 with age, resulting in prominent changes in global levels of H3 methyl modifications [27]. In addition, H2A.Z has been reported to accumulate in the mouse hippocampus with age [28]. Another histone variant, H2A.J, accumulates in senescent human fibroblasts, upregulating the expression of inflammatory genes. It was also observed that the accumulation of H2A.J occurs in mouse hair follicle stem cells, mouse interfollicular epidermal cells, and human epidermal cells with age [29]. Thus, the replacement of canonical histones with histone variants is likely to be a common feature in aging. Additionally, histone chaperones affect nucleosome composition through deposition of histones on DNA or eviction of histones from nucleosomes. Although HIRA, a histone chaperone, is reported to be required for the dynamics of the chromatin landscape, including H3.3 deposition and H4K16ac retention, in senescent cells [34], very little is known about the functional importance of histone chaperones for histone variants during aging. Thus, it may be interesting to explore the role of histone chaperones in aging.

### 2.3. ATP-Dependent Chromatin Remodeling

The chromatin landscape can be altered via nucleosome assembly, nucleosome repositioning, and exchange of histone variants by ATP-dependent chromatin remodelers, which play an important role in chromatin accessibility [35]. Chromatin remodelers are divided into four major subfamilies: imitation switch (ISWI), chromodomain helicase DNA-binding (CHD), switch/sucrose non-fermentable (SWI/SNF), and INO80 [36]. Several chromatin remodeling complexes have been reported to be associated with age-associated chromatin remodeling and regulation of lifespan (Table 1). It was previously reported that deletion of ISW2 causes nucleosome positioning changes at 1187 gene loci across the genome in yeast [37]. Interestingly, Dang and coauthors revealed that deletion of ISW2, which encodes the catalytic component of the ISW2 complex, promotes longevity through induction of genotoxic stress response, which partially mimics calorie restriction effects. These results suggest that ISW2 negatively modulates longevity through stress response pathways in yeast [31]. In *Caenorhabditis elegans*, the distinct chromatin remodeler SWI/SNF is linked to expansion of lifespan. SWI/SNF as a cofactor colocalizes with DAF-16/FOXO at target promoters to activate transcription. Inactivation of the SWI/SNF by RNAi reduced lifespan and stress resistance mediated by DAF-16/FOXO [30]. Another study showed that Mi2 (CHD-3/CHD-4) plays a negative regulator role in longevity. For example, mutations in LET-418, an Mi2 homolog in *C. elegans,* extend lifespan and enhance stress resistance in a DAF-16/FOXO-dependent manner [32]. Thus, ATP-dependent chromatin remodeling factors function as a positive and negative regulator of longevity by controlling stress resistance.

### 2.4. Histone Methylation

Histone methylation contributes to either transcriptional activation or transcriptional repression, depending on the histone, the residue modified, and the level of methylation. Generally, methylation of H3K4, H3K36, and H3K79 is linked to active transcription, whereas that of H3K9, H3K27, and H4K20 is associated with repressive transcription [38]. This modification can be added by histone methyltransferases and removed by histone demethylases [39]. A large number of studies have reported that histone methylations modified by histone methyltransferases or histone demethylases change during aging (Table 2).

Heterochromatin remains condensed and transcriptionally silent. Heterochromatin is known to be associated with H3K9me3, H4K20me3, and H3K64me3 in addition to DNA methylation [55,56,57]. The heterochromatin loss model of aging proposes that heterochromatin domains established early in embryogenesis are decreased during the aging process, contributing to the global loss of heterochromatin-induced gene silencing and leading to aberrant gene expression patterns [58]. Global heterochromatin loss with aging has been reported in humans and several model organisms [59,60,61,62,63]. Models of premature aging diseases, such as Hutchinson–Gilford progeria syndrome (HGPS) and Werner syndrome, exhibit heterochromatin loss with a decrease of H3K9me3, HP1, or SUV39H, a H3K9me3 histone methyltransferase [62,64]. For example, HGPS is caused by germline mutations in the *LMNA* gene, leading to the expression of progerin, a truncated mutant form of lamin A. Cultured cells from HGPS patients show abnormal nuclear morphology, including enlarged nuclei and heterochromatin loss with decreased H3K9me3 and reduced HP1 expression [62,65,66]. Although heterochromatin loss is observed during senescence, localized heterochromatin referred to as senescence-associated heterochromatin foci (SAHFs) has also been reported in senescent cells [67,68,69,70]. Recently, this contradictory role of heterochromatin in cellular aging and SAHF formation was further investigated through Hi-C analysis [71]. It was observed that HGPS cells show a loss of local internal structure in heterochromatin but do not exhibit the spatial clustering of heterochromatin. In contrast, in senescent cells, a loss of internal structure in heterochromatic regions is accompanied by spatial clustering of the heterochromatic regions. These findings suggest that the senescence-specific spatial clustering of heterochromatic regions may be required for SAHF formation. Taken together, these studies indicate that higher order chromatin structures are altered during aging.

#### 2.4.1. H3K4me3

H3K4me3, an active histone mark, is most abundant around transcription start sites [38]. There are several reports of the use of targeted RNAi to show that modifying H3K4me3 enzymes can regulate the lifespan of flies and worms (Table 2). Greer et al. reported that deficiencies in ASH-2, WDR-5, and SET-2, components of the ASH-2 Trithorax complex, cause a global decrease in H3K4me3 levels and extend lifespan in fertile worms. Meanwhile, knockdown of RBR-2, an H3K4me3 demethylase, increases H3K4me3 level and shortens lifespan, counteracting the effect of the ASH-2 methyltransferase complex. Overexpressing RBR-2 in the germ line extends lifespan [49]. Consistent with this, a deficiency in Lid, the ortholog of RBR-2, decreases lifespan and increases H3K4me3 levels in male flies, although it has no effect on the lifespan of female flies [50]. However, a deficiency in Trithorax, a component of another type of H3K4me3 methyltransferase complex in *Drosophila*, has no significant effect on the lifespan of male flies [43]. It seems, therefore, that regulators of H3K4me3 have different effects on lifespan depending on the H3K4me3 complex or organism.

#### 2.4.2. H3K9me3

H3K9me3, a repressive mark, is known to be associated with heterochromatin. There are some reports that implicate H3K9me3-modifying enzymes in aging. In *Drosophila*, deletion of Kdm4A, an H3K9me3 demethylase, was observed to affect fly lifespan. The disruption of Kdm4A shortens the male lifespan, suggesting that KDM4A plays a role in longevity [51]. Another report demonstrated that expression of the histone methyltransferase SUV39H1 in both human and mouse hematopoietic stem cells (HSCs) decreased concomitant with age, leading to a global reduction in H3K9me3 and perturbed heterochromatin function [40]. The authors further found that expression of the microRNA miR-125b, a known regulator of HSC function, increased with age in human HSCs. Since miR-125b directly targets SUV39H1, overexpression of miR-125b caused changes in heterochromatin structure and loss of B cell potential, indicating that an age-dependent decrease in SUV39H1 is closely associated with the destruction of heterochromatin structure and B lymphocyte formation in HSCs. These studies suggest that global changes in H3K9me3 are accompanied by heterochromatin misregulation, affecting lifespan depending on organism.

#### 2.4.3. H3K27me3

The trimethylated histone H3 at lysine 27 (H3K27me3) is likely to be modified dynamically during aging. H3K27me3 denotes transcriptional silencing, which is produced by polycomb repressive complex-2 (PRC2) and functionally maintained by PRC1 [72]. PRC2 contains the PcG proteins E(Z), SU(Z)12, ESC (or ESCL), and PCL. E(Z), the catalytic subunit of PRC2, along with the other PRC2 subunits, catalyzes H3K27 trimethylation [73]. In *Drosophila*, mutation of E(Z), the *Drosophila* homolog of EZH1/2, and its H3-targeting partner protein ESC, increased longevity and reduced H3K27me3 levels [43]. A further study demonstrated that a reduction in H3K27me3 due to PRC deficiency promotes glycolysis and healthy lifespan [42]. Consistent with this, upregulation of the transcript levels of EZH1 and CBX7/8 and an increase in H3K27me3 were observed in the killifish brain during aging [44]. Additionally, the global level of H3K27me3 was increased in quiescent mouse muscle stem cells during chronological aging [21]. These results suggest that a decrease H3K27me3 extends lifespan in some animals.

On the contrary, reduction of H3K27me3 is associated with aging in other animal models. UTX/KDM6A has been identified as a demethylase for H3K27me3 [74,75]. By removing the transcriptionally repressive H3K27me3 mark, UTX can antagonize transcriptional repression via PRCs. A decrease in H3K27me3 and an increase in UTX-1 concomitant with aging were observed in *C. elegans* and humans [52,53,76]. RNA interference (RNAi) in the *utx-1* gene extends lifespan in *C. elegans* in a *daf-16*-dependent manner. Furthermore, it was observed that H3K27me3 on the *daf-2* gene, which encodes the insulin-like receptor DAF-2, is profoundly reduced in aged worms and that *utx-1* RNAi significantly increased the H3K27me3 level on the *daf-2* gene. These results suggest that UTX-1 plays an important role in lifespan in *C. elegans* via modulation of the insulin/IGF-1 signaling pathway. Additionally, Bracken et al. demonstrated that EZH2 is downregulated in stressed and senescent human lung cells, leading to the loss of H3K27me3 [41]. Although the results of studies on several models, including worms, flies, fish, and senescent cells, showed that the link between H3K27me3 levels and aging is complex, it is possible that depending on the specific loci and cells, different H3K27me3 regulators may influence lifespan. Thus, aging may be associated with both an increase and decrease in H3K27me3.

#### 2.4.4. H3K36me3

H3K36me3 is associated with transcriptional elongation and is located in gene bodies rather than promoters [77]. Recently, it was reported that H3K36 methylation plays an important role in transcriptional precision, affecting longevity. Mutation of H3 at the K36 residue and deletion of Set2, an H3K36 methyltransferase, shortened lifespan; deletion of the H3K36 demethylase, Rph1, increased H3K36me3 levels and extended lifespan in wild-type yeast but not H3K36 mutant yeast. These results indicate that methylation at H3K36 is required for lifespan extension. In addition, loss of sustained histone H3K36 methylation is associated both with increased cryptic transcription in a subset of genes in old cells and with decreased lifespan [45]. Consistent with this, Pu et al. revealed that knockdown of Met1 methyltransferase induces a decline in global H3K36me3 levels, resulting in an increase in mRNA expression change with age and shortened lifespan in *C. elegans* [46]. While there is no significant change in H3K36me3 distribution during aging in *C. elegans* somatic cells, H3K36me3 marking negatively correlates with changes in gene expression during aging in *C. elegans* as well as *Drosophila* [46]. These results suggest a conserved role for H3K36me3 marks in maintaining transcriptional consistency and longevity.

While decreased trimethylation of H3K36 in worms and yeast is known to be associated with reduced lifespan, the role of dimethylation of H3K36me2 in aging is yet to be clarified. Recently, *C. elegans* SET-18 was identified as a histone H3K36 dimethyltransferase. The deletion of SET-18 increased lifespan and oxidative stress resistance depending on *daf-16* activity in the insulin/IGF pathway. Muscle-specific expression of SET-18 increased in aged worms, resulting in elevation of global H3K36me2 and inhibition of *daf-16a* expression and, consequently, decreased longevity. These results suggest that H3K36me2 and H3K36me3 modification have distinct functions in regulating aging [47].

### 2.5. Histone Acetylation

An acetyl group can be added to the ε-amino group of a histone lysine, resulting in neutralization of the positive charge of the lysine and weakening of the interaction between the histone and DNA. The resultant decondensed chromatin structure leads to transcription activation [78,79]. Generally, histone acetyltransferases (HATs) are regarded as coactivators in transcription as they catalyze lysine acetylation and loosen chromatin structure, whereas histone deacetylases (HDACs) are considered to function as corepressors. HATs and HDACs are known to play a critical role in longevity (Table 3). For example, in *Drosophila*, an age-related decrease in H3K5 acetylation level is suppressed by dietary restriction but accelerated by RNAi of the histone acetyltransferase CBP-1 [80,81]. Recently, an interesting report suggested that CBP plays a role in modulating longevity in the pea aphid (*Acyrthosiphon pisum*), a laboratory insect model; RNAi of *A. pisum* p300/CBP reduces both lifespan and number of offspring, suggesting that the manipulation of CBP contributes to accelerated aging [82]. On the other hand, it was observed that deletion of the histone acetyltransferase gene GCN5 reduces the extension of replicative lifespan in yeast [83]. In summary, a series of histone acetyltransferases or deacetylases are involved in the process of aging.

#### 2.5.1. H3K9ac

In mice, a deficiency of SIRT6, a member of the sirtuin (SIRT) family of NAD^+^-dependent deacetylases, causes a decrease in lifespan and a premature aging-like phenotype [94]. SIRT6 is reported to function as an NAD^+^-dependent H3K9 deacetylase that modulates telomeric chromatin [98]. Notably, Kawahara et al. showed that SIRT6 deacetylates H3K9ac and blocks hyperactive NF-κB signaling, which may modulate lifespan of mice [93]. Consistent with the above mentioned studies, SIRT6 overexpression increased longevity in transgenic mice [95] and inhibited the senescence of rat and human nucleus pulposus cells in a model of intervertebral disc degeneration [96]. These results suggest that SIRT6 may contribute to longevity via H3K9ac deacetylation.

#### 2.5.2. H3K56ac

The acetylation of lysine 56 on histone H3 (H3K56ac) promotes de novo nucleosome assembly, genomic stability, transcription, and formation of heterochromatin/euchromatin boundaries [99,100,101]. In yeast, the level of H3K56 acetylation decreased with age, while the level of H4K16 acetylation increased [85]. Consistent with this, Feser et al. showed that deletion of Rtt109, a major acetyltransferase for H3K56, reduces lifespan in yeast [17]. Unexpectedly, they also found that lifespan is somewhat reduced by deletion of the H3K56-associated deacetylases Hst3 and Hst4. These contradictory results suggest that a delicate balance of acetylated H3 K56Ac may be required to promote longevity.

#### 2.5.3. H4K12ac

A recent report showed that during early aging in *Drosophila*, elevated acetyl-CoA levels cause changes in histone acetylation. Specifically, a reduction in the levels of the histone lysine acetyltransferase Chameau (HBO1, KAT7) decreases H4K12 acetylation and extends the lifespan of *Drosophila* males [84].

#### 2.5.4. H4K16ac

The sirtuin family, a group of NAD^+^-dependent deacetylases, has been strongly linked to increased lifespan [102,103]. Sir2, a member of the sirtuin family, was first identified in the budding yeast *Saccharomyces cerevisiae* and was found to remove H4K16ac and H3K56ac [104]. During replicative yeast aging, *sir2* mutants exhibit a shortened lifespan [88]. Furthermore, an age-associated decrease in Sir2 protein is coupled with an increase in H4K16 acetylation, resulting in compromised transcriptional silencing in subtelomeric regions [85]. In *C. elegans*, increased dosage of the *SIR2* ortholog sir-2.1 extends mean lifespan by up to 50% [89]. This extension requires the FOXO transcription factor DAF-16. Rogina et al. reported that an increase in *Drosophila* Sir2 (dSir2) extends lifespan [90]. Sas2 is the major H4K16 acetyltransferase that establishes boundaries between telomeres and euchromatin [105,106]. Deletion of Sas2, a histone acetylase, extends lifespan [85]. The antagonizing activities of Sir2 and Sas2 establish an H4K16ac gradient in close proximity to telomeres, maintaining a silencing boundary. In mammals, there are seven sirtuins (*SIRT1–SIRT7*) that exhibit different enzymatic activities and subcellular compartmentation [107]. While many reports have shown that SIRT1 and SIRT6 are involved in longevity (Table 3), it is a mystery whether H4K16ac is a main target of those SIRT proteins in mammalian aging.

Several reports have shown that H4K16 acetylation is somewhat decreased in an age-dependent manner in mammals. A deficiency of Zmpste24 induces accumulation of prelamin A, which interferes with the retention of MOF, a histone acetyltransferase of H4K16, inducing hypoacetylation of H4K16 and cellular senescence [97,108]. Furthermore, supplementation with a histone deacetylase inhibitor, sodium butyrate, extends the lifespan of Zmpste24-deficient mice [108]. Recent studies have demonstrated that aging in HSCs is correlated with a reduction in H4K16ac and loss of polar distribution of H4K16ac [109,110]. Therefore, the role of H4K16ac in regulating lifespan needs to be clarified.

#### 2.5.5. H4 N-Terminal Acetylation

Calorie restriction decreases the level of Nat4, which belongs to the N-terminal acetyltransferases family and shows high substrate selectivity for histone H4 and H2A. In yeast, Nat4 deletion and the loss of H4 N-terminal acetylation extend lifespan as part of a calorie restriction–mediated pathway [111]. These findings reveal that histone N-acH4 functions as a modulator of lifespan linked to calorie restriction to promote longevity.

### 2.6. Histone Phosphorylation

A recent report showed links between nutrition sensing pathways and chromatin regulation in aging. It was found that in yeast under nutritional stress, the level of histone H3 threonine 11 phosphorylation (H3pT11) is specifically increased at stress-responsive genes, regulating the transcription of genes involved in metabolic transition. Sch9 and Cka1, a catalytic subunit of CK2, are required for the phosphorylation of H3T11 under such stress [112]. These results suggest that H3pT11 functions as a marker of nutritional stress and aging.

In *Drosophila*, histone H3S28A mutants (mimicking H3S28 phosphorylation) are associated with increased longevity and improved resistance to starvation and paraquat-induced oxidative stress [113] Additionally, whole-exome deep gene sequencing has revealed that H3S28A mutants exhibit a differential expression pattern of longevity-promoting and mitochondrial biogenesis and respiration genes. These findings present a potential role for H3S28 phosphorylation in controlling longevity and stress resistance.

### 2.7. Histone Ubiquitination

Histone H2B monoubiquitination is required for the trimethylation of both H3K4 and H3K79 by COMPASS and Dot1 methyltransferases, respectively [114]. Interestingly, the accumulation of H2B monoubiquitination is observed at the telomere-proximal regions of replicative aged yeast, accompanied by an increase in H3K4me3, H3K79me3, and H4K16ac. Rhie et al. demonstrated that H2BK123 mutants have short lifespans in yeast; deletion of the components of the H2B ubiquitinase complex Rad6/Bre1 and the deubiquitinase Ubp10 decreases lifespan [115]. Furthermore, deletion of some components of the SAGA/SLIK histone deubiquitinase complex, including Sgf73, Ubp8, and Sgf11, extends lifespan in yeast [116]. Collectively, an increase of H2B ubiquitination appears to be linked to replicative aging. However, the role of H2B ubiquitination in aging is yet to be investigated.

### 2.8. Genome-Wide Profiles of Chromatin Accessibility and Histone Modifications during Aging

Comparative chromatin analysis of young cells and old cells reveals changes in their gene expression patterns, an increase in variation in gene expression, and, further, elevated cell-to-cell variability. Recent technological breakthroughs, such as ChIP-seq, ATAC-seq, and RNA-seq, have allowed researchers to investigate genome-wide chromatin dynamics using a small number of cells or even single cells [117].

#### 2.8.1. Chromatin Immunoprecipitation followed by Sequencing (ChIP-seq) and RNA-seq

A recent study revealed that depletion of lamin B1 triggers disruption of nuclear lamin interaction and causes large-scale changes in the chromatin landscape during senescence [54]. The authors found that relative levels of H3K4me3 and H3K27me3 normalized to H3 do not differ between proliferating and senescent cells. Interestingly, H3K4me3 and H3K27me3 marks were redistributed during senescence. The formation of K4me3-enriched domains and K27me3-enriched domains occurs within lamin-associated domains. H3K27me3-depleted regions often contain enhancers near key senescence genes, and the H3K27me3 loss is associated with the upregulation of senescence gene expression. In addition, the distribution of H4K20me3 in senescent cells was determined using ChIP-seq and immunofluorescence; H4K20me3 was enriched at SAHFs, and this was linked with the presence of H3K9me3 and the depletion of H4K16ac and DNA methylation [118].

Genome-wide ChIP-seq and RNA-seq analyses have revealed that downregulation of lamin B1 during senescence triggers global and local chromatin changes that impact gene expression and aging. Other ChIP-seq data shows a unique pattern of H3K4me3 changes during aging in *C. elegans* [119]. It was shown that around 30% of H3K4me3-enriched regions exhibit significant and reproducible changes with age. These dynamic H3K4me3 regions are largely assigned to protein coding genes and are enriched in areas that span the gene body, where they lead to changes in gene expression. Sun et al. performed the first extensive study integrating epigenomic information on multiple histone modifications and DNA methylation in young and old HSCs. ChIP-seq with H3K4me3 and H3K27me3 has shown that old HSCs exhibit a 6.3% increase in the number of H3K4me3 peaks and broader H3K4me3 peaks across genes related to HSC identity and self-renewal. H3K27me3 peak counts in aged HSCs are similar to those in young HSCs, but the peaks show increased length of coverage. The authors also examined bivalent domains with H3K4me3 and H3K27me3; 335 bivalent domains disappeared in aged HSCs, while 1245 bivalent domains appeared [120]. These age-associated changes in histone modifications may contribute to functional changes in aged HSCs. Most recently, Benayoun et al. generated chromatin maps and transcriptomes from four tissues and one cell type from young, middle-aged, and old mice. The results showed general rules and patterns in age-related chromatin and transcriptional changes with age [121]. Machine-learning analysis with potential predictors revealed that age-related epigenomic changes can predict transcriptional changes and that immune responses are upregulated during aging across tissues and species. These comprehensive findings are likely to aid in the identification of strategies to control aging and age-related diseases.

#### 2.8.2. Assay for Transposase-Accessible Chromatin Using Sequencing and RNA-seq

Moskowitz et al. published genome-wide maps of chromatin accessibility in purified CD8+ T cells from human young and old donors [122]. Integrated assay for transposase-accessible chromatin using sequencing (ATAC-seq) and transcriptome analysis have shown that naïve and central memory cells from older individuals genetically shift toward more differentiated patterns of chromatin openness. In addition, aged naïve cells displayed a loss in chromatin accessibility at gene promoters, largely associated with a decrease in nuclear respiratory factor 1 (NRF1) binding, resulting in lower metabolic activity in aged T cells. Through combined analysis of chromatin accessibility and the transcriptome, Ucar et al. showed the epigenomic signature of aging in peripheral blood mononuclear cells (PBMCs) with chromatin closing at promoters and enhancers and chromatin opening at quiescent and repressed sites. The authors also defined the chromatin accessibility signature of aging-related reduced immune responses in PBMCs. The chromatin accessibility profiles of purified cells show that this aging-related signature in PBMCs stems from CD8+ T cells, which account for 10%–15% of PBMCs [123]. Most recently, Koohy and coauthors performed RNA-seq, ChIP-seq, ATAT-seq, and Hi-C to examine how gene expression, chromatin states, and genome organization are altered in developing B cells during mice aging. The authors observed that aging affected the expression of a limited number of genes and identified transcriptional downregulation of insulin-like growth factor signaling pathway as a hallmark of aging in the aged B cell precursors. These changes are accompanied by changes in microRNAs, histone modifications, and higher-order chromatin structure. It is also found that active gene expression is more strongly linked to H3K4me3 and H3K27ac than to chromatin accessibility, suggesting the combined analysis of the epigenome and transcriptome provides more detail and genome-wide information [124].

#### 2.8.3. Single-Cell Approach

Single-cell RNA sequencing and single-nucleus RNA sequencing have emerged as a powerful tool to reveal the cellular complexity and heterogeneity during aging [125,126,127]. However, epigenetic changes at single-cell resolution during aging remain largely unknown. Recently, Cheung et al. developed a highly multiplexed mass cytometry analysis to profile the global levels of a number of chromatin modifications in human immune cells at the single-cell level [117]. Comparative single-cell ChIP analysis of younger and older adults indicated increased variation between individuals and elevated cell-to-cell variability in chromatin marks as signatures of aging. Through analysis of twin pairs, the authors identified divergent chromatin modification profiles in older monozygotic twin pairs compared to younger pairs, suggesting that non-heritable factors contribute considerably to increased variation in chromatin marks with age.

## 3. Conclusions and Future Perspectives

**Figure 1:**
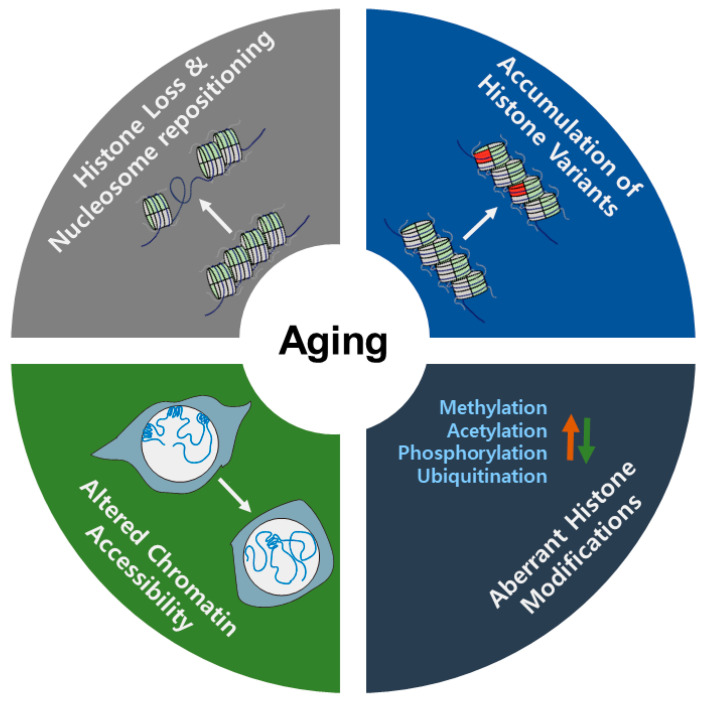
Changes in chromatin states linked to aging.

Over time, many researchers have focused on the mechanisms underlying aging. The aging process is regulated by various internal (e.g., genomic instability, epigenetic changes, and metabolic dysfunction) and external (e.g., nutrients and exercise) factors. Epigenetic regulation is interconnected with other factors. During the aging process, epigenetic changes, including variation in histone levels, exchange of histone variants, histone modifications, and nucleosome occupancy, are dynamically changed, modulating chromatin states (see Figure 1).

In this review, we provided comprehensive information on the roles of epigenetic modifications in aging. In general, histone levels are decreased and aberrant nucleosome occupancies occur during aging. The accumulation of histone variants is another common feature in the aging process. In contrast, histone modifications such as acetylation, methylation, phosphorylation, and ubiquitylation are more complex. These modifications are differentially implicated in aging; some active (e.g., H3K9ac and H4K16ac) or repressive marks (e.g., H3K9me3 and H3K27me3) may be globally increased or decreased, respectively, during the aging process. In contrast, the active mark H3K36me3 is generally decreased in aging. In other cases, gene-specific changes in histone modifications (e.g., H3K27me3) modulate the expression of key genes related to longevity. Despite increased knowledge on these topics, many questions remain unanswered regarding the mechanism of the effects of epigenetic marks on longevity. Although genetic studies using loss of function have revealed the biological significance of histone modifications in aging, we need to extend our understanding of exactly how these modifications regulate the aging process. Several recent technological advances provide promising clues that may help us to tackle these challenges. For example, ChIP-seq and ATAC-seq combined with transcriptome analysis from the tissue to the single cell level, provide novel findings. As discussed in this review, age-related epigenetic marks are somewhat different between invertebrates and vertebrates. The epigenetic mechanisms of aging are more complicated in vertebrates due to their specialized systems. The development of new experimental models for aging research will provide key insights into the longevity and epigenetic pathways that underlie aging. Indeed, a better understanding of the epigenetic mechanisms of aging and longevity will help us to develop innovative therapeutics that will improve lifespan and counter aging.

## Figures and Tables

**Table 1:** Modulation of histones and chromatin remodelers during aging.

Name	Description	Epigenetic Changes Linked to Aging	Effect on Longevity/Health Span	Model	Reference
Histones					
H3, H4, H2A, H2B	Canonical histones	Canonical histone loss	+	Yeast	[17,18]
H3, H4	Canonical histones	Canonical histone loss	+	Human senescent cell	[19,20]
H2A.Z and H3.3	Histone variants	Accumulation of H2A.Z and H3.3	−	Human senescent cell	[24]
MacroH2A	Histone variant	Increased protein level of macroH2A	−	Senescent cell, tissues of mice and primates	[25]
H3.3	Histone variant	Accumulation of H3.3	−	Mouse neuron and glial cells	[26]
H3.3	Histone variant	Accumulation of H3.3	−	Various mouse somatic tissues	[27]
H2A.Z	Histone variant	Accumulation of H2A.Z	−	Mouse hippocampus	[28]
H2A.J	Histone variant	Accumulation of H2A.J	−	Senescent human fibroblasts, mouse hair follicle stem and interfollicular epidermal cells, human epidermis	[29]
**Chromatin Remodelers**					
SWI/SNF	ATP-dependent chromatin remodeler	SWI/SNF is required for DAF-16/FOXO-mediated dauer formation, stress resistance and longevity	+	*Caenorhabditis elegans*	[30]
Isw2 and Itch	ATP-dependent chromatin remodeler		−	Yeast	[31]
LET-418	Mi2 homolog (NurD complex)		−	*C. elegans*	[32]

+: positive, −: negative.

**Table 2:** Histone methylation-modulating proteins and aging.

Name	Description	Change in Histone Modifications Linked to Aging	Effect on Longevity/Health Span	Model	Reference
Methyltransferases					
SET-2	Histone lysine methyltransferase (ASH-2 complex subunit)	H3K4me3 ↑	−	*C. elegans*	[38]
ASH-2	ASH-2 complex subunit				
WDR-5	ASH-2 complex subunit				
SUV39H1	Histone methyltransferase	H3K9me3 ↓	+	Human and mouse HSCs	[40]
EZH2	Methyltransferase	H3K27me3 ↓	+	Human senescent cell	[41]
E(Z)	Methyltransferase (PRC2 complex subunit)	H3K27me3 ↑	−	*Drosophila*	[42,43]
ESC	PRC2 complex subunit				
EZH1, CBXs	Polycomb complex	H3K27me3 ↑	−	Killifish brain	[44]
Set-2	Histone lysine methyltransferase	H3K36me3 ↓	+	Yeast	[45]
MET-1	Histone lysine methyltransferase	H3K36me3 ↓	+	*C. elegans*	[46]
SET-18	H3K36 di-methyltransferase	H3K36me2 ↑	−	*C. elegans*	[47]
**Demethylases**					
T08D10.2	H3K4me/me2 demethylase, LSD-1 ortholog		−	*C. elegans*	[48]
RBR-2	H3K4me3 demethylase	H3K4me3 ↑	+	*C. elegans*	[49]
Lid	Demethylase, RBR-2 ortholog	H3K4me3 ↑	+	*Drosophila*	[50]
KDM4A	Histone demethylase	H3K9me3 ↑	+	Male *Drosophila*	[51]
UTX-1	H3K27me3 histone demethylase	H3K27me3 ↓	−	*C. elegans*	[52,53]
RPH1	H3K36me2/3 demethylase	H3K36me3 ↓	−	Yeast	[45]
**Others**					
HP1 beta	Methylated histone reader, heterochromatin marker		−	Senescent cell, tissues of mice and primates	[25]
Lamin B1	A component of nuclear lamina	H3K27me3 ↓	+	Human senescent cell	[54]

↑: increase, ↓: decrease, +: positive, −: negative.

**Table 3:** Histone acetylation-modulating proteins and aging.

Name	Description	Change in Histone Modifications Linked to Aging	Effect on Longevity/Health Span	Model	Reference
Acetyltransferases					
Gcn5	Histone acetyltransferase		+	Yeast	[83]
CBP	Histone acetyltransferase	H4K5ac ↓	+	*C. elegans*	[81]
CBP	Histone acetyltransferase		+	*Acyrthosiphon pisum*	[82]
Chameau	MYST domain acetyltransferase, human HBO1 homolog	H4K12ac ↑	−	*Drosophila*	[84]
Sas2	Histone acetyltransferase	H4K16ac ↑	−	Yeast	[85]
Asf1	Histone chaperone	H3K56ac ↓	+	Yeast	[17]
RTT109	Histone acetyltransferase	H3K56ac ↓			
**Deacetylases**					
Rpd3	Histone deacetylase, HDAC1 ortholog		−	Yeast; *Drosophila*	[86,87]
Sir2	NAD^+^-dependent deacetylase	H4K16ac ↑	+	Yeast	[85,88]
Sir-2.1	Sir2 ortholog		+	*C. elegans*	[89]
dSir2	Sir2 ortholog		+	*Drosophila*	[90]
SIRT1	NAD^+^-dependent deacetylase		+	Mice	[91]
SIRT1	NAD^+^-dependent deacetylase		+	Human and mouse fibroblast senescent cells	[92]
SIRT6	NAD^+^-dependent deacetylase	H3K9ac ↑	+	Mice	[93,94]
SIRT6	NAD^+^-dependent deacetylase		+	Mice	[95]
SIRT6	NAD^+^-dependent deacetylase		+	Rat and human nucleus pulposus senescent cells	[96]
**Others**					
ZMPSTE24	Lamin A processing protease	Acetylation of H4 and H2B ↑	+	Premature aging mouse model	[97]
HIRA	Histone chaperone	H4K16ac retention	−	Human senescent cells	[34]

↑: increase, ↓: decrease, +: positive, -: negative.
